# Drinking Patterns and the Association between Socio-Demographic Factors and Adolescents’ Alcohol Use in Three Metropolises in China

**DOI:** 10.3390/ijerph120202037

**Published:** 2015-02-12

**Authors:** Shijun Lu, Songming Du, Xiaoqi Hu, Shurong Zou, Weijia Liu, Lei Ba, Guansheng Ma

**Affiliations:** 1 National Institute for Nutrition and Health, Chinese Center for Disease Control and Prevention, Xicheng District, Beijing 100050, China; E-Mails: lushijun666666@163.com (S.L.); dusm9709@126.com (S.D.); huxiaoqee@163.com (X.H.); 2 Shanghai Municipal Center for Disease Control and Prevention, Changning District, Shanghai 200336, China; E-Mail: zoushurong@scdc.sh.cn; 3 Guangzhou Center for Disease Control and Prevention, Baiyun District, Guangzhou 510440, China; E-Mail: liuweijia88@hotmail.com; 4 Beijing Chaoyang District Center for Disease and Prevention, Chaoyang District, Beijing 100021, China; E-Mail: baleibalei@126.com

**Keywords:** adolescent, alcohol use, drinking pattern, socio-demographic factors

## Abstract

The current study was designed to investigate the drinking patterns and association between socio-demographic factors and adolescents’ alcohol use among high school students from China’s three metropolises, Beijing, Shanghai, and Guangzhou. Using a self-administered questionnaire, we conducted a cross-sectional survey among 13,811 high school students from 136 schools between May and June 2013. A two-stage stratified sampling method was used for subject selection. The prevalence of lifetime drinking was 52.5%; in addition, 38.5% of the students were past-year drinkers, while 20.1% of them had consumed alcohol in the past 30 days. During the past year, 29.7% of the students reported that they drank once per month or less, and 22.0% of the students drank less than one standard drink (SD) per occasion. For the students who were not living with their mothers, as well as the students in higher socioeconomic status (SES), the adjusted odds of past and current drinking were significantly higher, compared with those who lived with both parents and low SES. Due to the high prevalence of alcohol consumption among junior and senior high school students in metropolises, attention should be paid by parents, school administrators, educational and public health agencies for making efforts collectively to reduce alcohol availability and drinking among adolescents.

## 1. Introduction

Adolescent drinking continues to be a significant social and public health issue worldwide [1]. It has been shown that adolescent drinking is associated with abnormalities in brain functioning, including memory deficits and poor academic performance [2,3,4,5]. In addition, adolescent drinking brings about many health risks, such as drunk driving, violence, illicit drug use, unsafe sex, and suicide [6,7,8,9]. Thus, during the past few years, numerous attempts have been developed for preventing and controlling of alcohol-related problems among youth [10].

To develop appropriate interventions, it is essential first to understand the nature and the trend of adolescent drinking within a given cultural context. Many countries have carried out periodic surveillance activities to monitor drinking behavior among youth [11,12]. Although China has century-long history of alcohol use [13] as well as a large population of adolescent [14], the population data of adolescent alcohol use in multi-metropolises as well as its correlates during the past decades are surprisingly limited.

In addition to estimating the prevalence of alcohol use among teenagers, the accurate measurement in quantity and drinking patterns (particularly heavy episodic or binge drinking) are necessary. Usually, a “standard drink” (SD) is considered as optimum unit for analysis, to estimate true quantity of alcohol consumption [15,16]. To facilitate the use of SD measure, exact descriptions for converting different alcoholic beverage products and serving sizes into corresponding SDs have been developed [17]. However, there is no SD definition being developed, whereas a uniform method for estimating alcohol consumption has not been reported in China yet.

Previous studies showed evidence of the association between social-demographic factors and the adolescent drinking behavior. For example, compared to living with two-biological parents, teenagers living with other family members tend to be associated with a wide variety of problematic behaviors [18,19]. Similarly, adolescents with a downward socioeconomic mobility during childhood were more likely to use alcohol [20]. Thus, increasing divorce/separation rate [21] and family income [22] become hot topics among current Chinese public; therefore, it is crucial to examine the relationship between specific socio-demographic factors and adolescents’ drinking behavior.

The current study was designed to examine: (1) Currentprevalence and patterns of alcohol use; and (2) the association of alcohol use among these adolescents with socio-demographic variables. This information could help inform future policy regarding alcohol use among youth in China.

## 2. Methods

### 2.1. Sampling Methods and Recruitment

A cross-sectional survey with a two-stage stratified sampling approach was used in this study. Four administrative districts from each city, Beijing, Shanghai, and Guangzhou, (4/16, 4/16, and 4/12) were selected for the survey. Subsequently, in each district, we selected subjects from junior and senior high schools (nearly 1:1, based on the total number of junior and senior students). The senior high school includes regular and vocational senior high school (nearly 1:1, based on the total number of students in the two kinds of schools). Totally, 136 schools were selected in the 12 districts, and the number of the sampling students in each district based on the registered number of students. In China, both junior and senior high schools have three grades. Due to the entrance examinations for college or internship during the period of the survey, students in the third year were unable to complete the survey. Thus, our study sample was comprised of two grades (7th and 8th grades in junior high school, 10th and 11th grades in senior high school) of each school. The average class sizes were 25–60 students per class, and one regular class was selected in each grade. The study protocol was approved by the Ethics Committee for Research in Human Subjects of the National Institute for Nutrition and Food Safety, Chinese Center for Disease Control and Prevention, and written informed consent was given before the survey, and obtained from all participating students and their guardians.

All students in the selected classrooms were informed to complete a self-administered questionnaire that is voluntary, and anonymous during a regular class period (approximately 40 min). Students selected for the survey were informed about the objectives of the study, and had the right to refuse to participate without any consequences on their lives or study in school. The students who finished the questionnaire could get a mechanical pencil. Among all the students recruited, only a small proportion of them declined to complete the questionnaire, thus the total response rate of 96.5% in individual level. In all, no school refused to join in the survey, and the response rate was 100%. Data collection was undertaken from May to June 2013 during the second semester of the academic year in China.

### 2.2. The Questionnaire

The questionnaire used in this study was developed based on three questionnaires reported previously, *i.e.*, the alcohol section of the questionnaire used China’s National Nutrition and Health Survey in 2002 [23], the Global School-based Student Health Survey (GSHS) core questionnaire modules in China [24], and the Youth Risk Behavior Survey (YRBS) questionnaire in the United States [11]. These questionnaires were modified by adding items including demographic characteristics (e.g., gender, age) and drinking pattern (frequency of drinking, amount of alcohol per occasion). The questionnaire includes of the following sections: (1) socio-demographic information, (2) drinking behavior and patterns. Specifically, the section on drinking behavior and patterns included detailed questions on lifetime drinking history, drinking frequency and quantity, heavy episodic or binge drinking, and typical number of drinks per occasion.

Prior to the implementation, this questionnaire was reviewed by an expert panel representing key professional fields, including psychiatrists, psychologists, school teachers and administrators, senior alcohol researchers, and health promotion specialists. The questionnaire was then pilot-tested in three schools in Beijing that targeted each grade of junior and senior high students; the survey was judged to be feasible and acceptable.

### 2.3. Study Measures and Definitions

In the present study, we have several definitions for various drinkers. For a lifetime drinker, it was defined as a student who had consumed any alcohol previously, regardless of the quantity of alcohol consumed; for a past-year drinker, as a student who had consumed alcohol during the past one year before the survey; for a current drinker, we defined it as a student who consumed alcohol during the past 30 days, and for those students who drink at least 6 drinks (equal to 60 grams pure alcohol) on an occasion, they will be classified as heavy episodic or binge drinkers based on their immediate past 30-day drinking history. Drinking until intoxication was defined as the drinkers who show slurred speech, talkative or diminished senses, such as speaks louder, inability to hear and see clearly, glassy, unfocused eyes, or slowed mental processing (forgetting things, or losing their train of thought, cannot listen well, follow conversations well, or understand what others are saying). These behaviors usually told by the people drinking together.

We then specified that one SD contains 10 grams of pure alcohol, which corresponded to one can (330 mL) of beer, one small glass (25 mL) of moderate distilled wine, one glass (100 mL) of grape wine, one glass (80 mL) of yellow wine, or one serving (30 mL) liquor. To aid with the estimation of SD while completing the questionnaire, each survey respondent will be provided with a chart printed in color for describing a variety of common alcoholic beverages with their corresponding SD equivalents.

The family affluence scale (FAS) was used as an alternative measurement of family socio-economic status (SES). The final FAS score was calculated by adding the responses to four indicators; does your family own a car (0, 1, 2, or more); how many times did you travel away on holiday with your family during the past 12 months (0, 1, 2, 3 or more); do you have your own bedroom for yourself (0, 1 representing no, yes); and how many computers does your family own (0, 1, 2, 3, or more), which ranged from 0 to 9. The FAS scores were subsequently recoded into tertiles (high, middle, low socio-economic status, SES) within each district. This measure of socio-economic status was selected as previous research has demonstrated that students’ report on the component items of the FAS are in agreement with reports of their parents [25,26].

### 2.4. Statistical Analysis

The primary sampling unit was the district and the secondary unit was the school. The sampling probability at the class level was not taken into account because in different types of schools, class-setting rules are not the same. The sampling weight for the two-stage survey was calculated by multiplication of the total number of districts in the developed and geographic area divided by the number of selected districts and the total number of students in the selected district divided by the number of sampled students in that district. The number of districts in the three metropolises and the number of junior and senior high school students in each selected district were used as finite population corrections. Data on the students in these districts were obtained from the local education department.

Weighted means, percentages and standard errors (SE) for sample characteristics by school type were calculated. Weighted percentages for lifetime, past year, past 30 day, binge drinking, drinking to intoxication and frequency and usual quantity of alcohol were calculated for the entire sample by school type, and compared between males and females using the Rao–Scott Chi-Square test. To evaluate associations between socio-demographic factors and drinking behavior among students, adjusted odds ratios (aORs) and 95% confidence intervals (CI), adjusted for all other variables in the model, were calculated using multiple logistic regression.

Subjects with missing values were removed from the analysis on a case-by-case basis in each analysis. Statistical analyses were conducted using SAS software V.9.3.

### 2.5. Quality Control

In this study, a standard training manual had been provided prior to the survey, accompanied by lecture presentations to explain the survey aim and the visual aid for estimating SDs. The data were collected by educators, doctors, and staff from the local Centers for Disease Control and Prevention, who were experienced in epidemiological surveys. Questionnaires were completed by students using scannable answer sheets. At the data entering stage, an automatic laser reader was used to scan the answer sheets, and double entry was performed and compared for identifying discrepancies. Finally, the optical scanning machine that connected with a computer, could convey the data in an excel file.

## 3. Results

Among the 136 selected schools, 67 were junior high schools, and 69 were senior high schools, including 36 regular senior high schools and 33 that were vocational senior high schools. The total number of students was 13,811 (49.7% males, 50.3% females), with 6575 and 7236 students in junior and senior high schools, respectively. The average age of all students was 15.3. The socio-demographic characteristics of the sample are listed in Table 1, which were similar to the range for general characteristics of high school students in China.

As shown in Table 2, the prevalence of lifetime drinking was 52.5%; 38.5% of the students were past-year drinkers, 20.1% had alcohol in their past 30 days, 4.8% had reached the binge drinking level, and 15.0% reported drinking until intoxication. The prevalence of drinking among male students for the five categories of consumption (56.3%, 43.5%, 23.8%, 6.7%, and 17.8%, respectively) was significantly higher in each category than the prevalence of drinking among female students (48.9%, 33.4%, 16.6%, 2.8%, and 12.3%, respectively).

Alcohol use increased with grade level in all five categories of consumption (Table 2). Lifetime alcohol use increased from 44.1% to 64.7%; drinking in the past 12 months increased from 30.5% to 50.1%; current drinking increased from 16.0% to 26.0%; binge drinking increased from 3.1% to 7.2%; and drinking to intoxication increased from 10.3% to 21.9% in junior high *vs.* senior high school students. In senior high school, the prevalence of drinking in the vocational senior high school was higher than for their counterparts in regular senior high school.

**Table 1:** Sample characteristics.

Characteristics	Junior High School (%SE^a^)		Senior High School (%SE^a^)				Total Number^b^
	G7 (*n* = 2821)	G8 (*n* = 3754)	G10^r^ (*n* = 1790)	G11^r^ (*n* = 1873)	G10^v^ (*n* = 1711)	G11^v^ (*n* = 1862)	
**Age**							
Mean (*S*^c^)	13.2 (0.91)	14.2 (0.76)	16.1 (0.70)	17.0 (0.79)	16.6 (1.09)	17.2 (1.03)	13,811
**Gender**							
Male	50.6 (1.33)	50.6 (1.06)	50.6 (1.28)	48.7 (2.01)	48.2 (3.64)	45.5 (5.63)	6726
Female	49.4 (1.33)	49.4 (1.06)	49.4 (1.28)	51.3 (2.01)	51.8 (3.64)	54.5 (5.63)	7085
**Living with:**							
Parents	82.3 (1.36)	82.9 (1.02)	79.1 (1.26)	79.3 (0.92)	72.3 (2.78)	75.9 (1.42)	10,936
Mother	9.2 (0.65)	7.5 (0.61)	9.2 (0.83)	8.9 (0.84)	9.1 (1.25)	8.8 (0.53)	1180
Father	3.2 (0.40)	3.4 (0.29)	2.5 (0.48)	2.2 (0.42)	3.9 (0.53)	5.0 (0.59)	426
Others	5.3 (0.79)	6.3 (0.81)	9.2 (1.10)	9.6 (1.04)	14.6 (2.47)	10.3 (1.37)	1,269
**SES**							
Low	19.5 (2.04)	19.7 (2.15)	16.9 (1.26)	17.5 (1.14)	30.3 (4.55)	25.6 (1.31)	2729
Middle	50.6 (1.26)	51.1 (1.05)	55.3 (1.85)	57.8 (1.35)	51.5 (2.88)	52.7 (1.48)	7031
High	30.0 (2.00)	29.2 (2.06)	27.8 (2.58)	24.7 (1.57)	18.2 (2.62)	21.7 (1.61)	3619

^a^ Based on the weighted data. ^b^ Based on the unweighted data, excluding missing data. ^c^ Indicates the standard deviation. ^r^ Indicates the regular senior high school. ^v^ Indicates the vocational senior high school.

**Table 2:** Prevalence of alcohol consumption by school and gender.

Drinking Patterns		Junior High School, %SE^a^			Senior High School, %SE^a^					Total
		G7	G8	Total	G10^r^	G11^r^	G10^v^	G11^v^	Total	
Lifetime drinking (*n*^b^ = 7075)	Male	42.9 (2.33)	52.0 (2.37)	48.0 (1.69)	61.6 (1.64)	64.3 (3.15)	75.2 (4.40)	78.5 (2.67)	68.5 (1.92)	56.3 (1.82)
	Female	36.8 (1.91)	42.7 (2.33)	40.1 (1.59)	49.2 (2.37)	53.4 (2.59)	71.1 (2.01)	76.9 (1.61)	61.1 (2.21)	48.9 (1.72)
	Total	39.8 (1.77)	47.4 (2.18)	44.1 (1.58)	55.5 (1.65)	58.7 (2.50)	73.1 (2.73)	77.6 (1.44)	64.7 (1.71)	52.5 (1.65)
	*p*^c^	0.010	<0.001	<0.001	<0.001	<0.001	0.321	0.638	0.002	<0.001
Drinking in the past 12 months (*n*^b^ =5119)	Male	29.0 (2.33)	38.3 (1.96)	34.1 (1.27)	51.1 (2.00)	52.7 (2.68)	62.6 (4.70)	68.4 (4.09)	57.3 (1.83)	43.5 (1.81)
	Female	22.5 (1.77)	30.1 (2.34)	26.8 (1.74)	35.7 (2.41)	36.0 (1.50)	51.2 (3.01)	56.0 (2.98)	43.0 (1.92)	33.4 (1.45)
	Total	25.8 (1.39)	34.2 (1.92)	30.5 (1.34)	43.9 (1.75)	44.2 (1.85)	56.8 (3.60)	62.0 (2.33)	50.1 (1.49)	38.5 (1.44)
	*p*^c^	0.033	<0.001	<0.001	<0.001	<0.001	0.001	0.026	<0.001	<0.001
Current drinking (*n*^b^ = 3010)	Male	15.8 (1.44)	20.1 (1.63)	18.2 (1.00)	27.7 (2.27)	25.1 (1.73)	40.3 (2.96)	41.7 (3.63)	32.1 (1.50)	23.8 (1.30)
	Female	12.2 (1.48)	15.2 (1.31)	13.9 (0.91)	14.7 (1.90)	11.9 (1.00)	28.0 (2.87)	32.3 (1.73)	20.3 (1.40)	16.6 (0.82)
	Total	14.0 (0.86)	17.6 (1.09)	16.0 (0.78)	21.2 (2.03)	18.3 (0.93)	34.0 (2.66)	36.6 (1.63)	26.0 (1.17)	20.1 (0.88)
	*p*^c^	0.130	0.013	<0.001	<0.001	<0.001	<0.001	0.033	<0.001	<0.001
Binge drinking (*n*^b^ = 761)	Male	3.8 (0.78)	4.4 (0.38)	4.1 (0.41)	6.4 (1.26)	6.2 (0.71)	16.3 (2.50)	18.3 (3.10)	10.7 (1.22)	6.7 (0.65)
	Female	1.3 (0.33)	2.5 (0.51)	2.0 (0.38)	1.6 (0.48)	1.4 (0.42)	6.9 (1.22)	7.6 (1.44)	3.9 (0.58)	2.8 (0.37)
	Total	2.6 (0.43)	3.5 (0.34)	3.1 (0.28)	4.0 (0.74)	3.8 (0.44)	11.4 (1.53)	12.4 (1.51)	7.2 (0.72)	4.8 (0.43)
	*p*^c^	<0.001	0.008	<0.001	<0.001	<0.001	<0.001	0.001	<0.001	<0.001
Drinking until intoxication (*n*^b^ = 2332)	Male	10.1 (0.87)	14.8 (1.36)	12.7 (0.93)	18.2 (1.11)	17.6 (2.03)	36.7 (4.30)	37.6 (3.70)	25.5 (1.99)	17.8 (1.21)
	Female	5.9 (0.50)	9.5 (1.15)	7.9 (0.67)	10.5 (1.33)	9.1 (1.23)	28.3 (2.81)	32.9 (3.31)	18.4 (1.95)	12.3 (1.07)
	Total	8.3 (0.41)	12.1 (1.04)	10.3 (0.64)	14.3 (0.85)	13.2 (1.34)	32.4 (3.15)	35.1 (2.69)	21.9 (1.77)	15.0 (1.03)
	*p*^c^	<0.001	<0.001	<0.001	<0.001	<0.001	0.014	0.291	<0.001	< 0.001

^a^ Based on the weighted data, excluding FAS missing data. ^b^ Indicates the total number of drinking students of that category. ^c^ Rao–Scott Chi-square tests were performed to examine the differences between males and females. ^r^ Indicates the regular senior high school. ^v^ Indicates the vocational senior high school.

The distribution of past-year frequency and quantity of alcohol use by gender is presented in Table 3. As with past and current history of alcohol use, frequency and quantity of alcohol consumption increased with grade level. Overall, during the past year, 29.7% of students at any grade level reported that they drank either once a month or less, 6.7% drank two to four times per month, and 2.0% drank two or more times weekly. With respect to quantity per occasion, among students at any grade level, 22.0% drank less than one drink, 11.4% consumed one to two drinks, 4.8% consumed three to six drinks, and 1.8% reached the maximum level of drinking of more than six drinks per occasion in the past year.

As shown in Table 4, the aORs for lifetime drinking (aOR = 0.72, 95% CI: 0.65–0.78), drinking in the past 30 days (aOR = 0.62, 95% CI: 0.54–0.71), and drinking in the past year (aOR = 0.64, 95% CI: 0.56–0.72) were lower among girls to boys. With the increase in grade level, students were more likely to report lifetime, past-year, or current drinking in comparison to younger students, after adjustment for covariates. This was especially true for vocational senior high school students where, for example, the 11th grade of vocational senior high school was associated with higher adjusted odds of drinking history (lifetime: aOR = 5.48, 95% CI: 4.37–6.88; past 30 days: aOR = 3.83, 95% CI: 3.16–4.71; past 12 months: aOR = 4.84, 95% CI: 3.79–6.17) than regular senior high school when compared to the youngest junior high students. Compared to students in Beijing, Shanghai’s students were more likely to consume alcohol in past-year (aOR = 1.49, 95% CI: 1.14–1.93) and Guangzhou’s students more likely to drink both in lifetime (aOR = 1.70, 95% CI: 1.40–2.08) and past-year (aOR = 1.98, 95% CI: 1.61–2.44). Students who did not live with their mothers, *i.e.*, living with their fathers or the other relatives were more likely to report history of drinking in all three categories as compared by living with both parents. Similarly, students in high SES were also more likely to report a history of alcohol in all three categories compared to low SES.

Results of further examination of the riskiest patterns of drinking, *i.e.*, binge drinking and drinking to intoxication, in association with socio-demographic factors is presented in Table 5. Females were less likely to engage in these drinking patterns (binge drinking: aOR = 0.39, 95% CI: 0.30–0.50; drinking to intoxication: aOR = 0.61, 95% CI: 0.53–0.69). From the junior high to senior high school students, higher-grade level was associated with riskier drinking patterns compared to younger students. As noted with history of alcohol, vocational senior high school students tend to drink in a risky pattern than regular senior high students compared to younger students. For example, the aOR for binge drinking in the overall senior high school second grade was 1.47 (95% CI: 0.97–2.22), whereas the aOR in the subset of those in the second grade of vocational high school was 6.01 (95% CI: 3.78–9.55). Students from Shanghai were less likely to involve in binge drinking (aOR = 0.62, 95% CI: 0.47–0.81) compared with Beijing. Students living with their fathers or living with the other relatives were significantly more likely to report binge drinking (fathers: aOR= 1.77, 95% CI: 1.20–2.62; others: aOR = 1.80, 95% CI: 1.38–2.36) and drinking until intoxication (fathers: aOR = 1.88, 95% CI: 1.42–2.49; others: aOR = 1.38, 95% CI: 1.21–1.69) in comparison to those who lived with both their parents. The high SES group tends to report binge drinking (aOR = 2.14, 95% CI: 1.58–2.88) and drinking until intoxication (aOR = 1.49, 95% CI: 1.26–1.76) in comparison to the low SES group.

**Table 3:** Drinking patterns during the past year by school and gender.

Patterns	Junior High School, % SE^a^				Senior High School, % SE^a^				Total, % SE^a^			
	Male	Female	Total	*p*^c^	Male	Female	Total	*p*^c^	Male	Female	Total	*p*^c^
**Frequency^b^**				<0.001				<0.001				<0.001
Did not drink last 12-months (*n* = 6693)	65.9 (1.27)	73.2 (1.74)	69.5 (1.34)	--	42.7 (1.83)	57.0 (1.92)	49.9 (1.49)	--	56.5 (1.81)	66.6 (1.45)	61.5 (1.44)	--
No more than once a month (*n* = 3916)	25.7 (1.03)	22.3 (1.36)	24.0 (1.08)	--	41.0 (1.41)	35.0 (1.61)	38.0 (1.20)	--	31.9 (1.37)	27.5 (1.09)	29.7 (1.10)	--
2–4 times a month (*n* = 916)	6.3 (0.99)	3.7 (0.46)	5.0 (0.49)	--	11.9 (0.81)	6.4 (0.69)	9.1 (0.60)	--	8.6 (0.71)	4.8 (0.43)	6.7 (0.42)	--
2–3 times a week (*n* = 191)	1.4 (0.29)	0.7 (0.18)	1.0 (0.20)	--	2.5 (0.39)	1.1 (0.19)	1.8 (0.16)	--	1.9 (0.25)	0.8 (0.14)	1.3 (0.15)	--
At least 4 times a week (*n* = 96)	0.7 (0.19)	0.1 (0.05)	0.4 (0.10)	--	1.8 (0.39)	0.5 (0.20)	1.2 (0.21)	--	1.1 (0.20)	0.3 (0.09)	0.7 (0.11)	--
**Usual quantity^b^**				<0.001				<0.001				<0.001
Non-drinker (*n* = 6840)	64.3 (1.36)	71.9 (1.66)	68.0 (1.33)	--	41.3 (1.75)	55.5 (1.79)	48.5 (1.40)	--	55.0 (1.85)	65.1 (1.38)	60.0 (1.43)	--
<1 SD^d^ (*n* = 2894)	21.8 (0.89)	18.5 (1.29)	20.2 (0.90)	--	23.7 (1.02)	25.6 (0.89)	24.7 (0.82)	--	22.6 (0.70)	21.4 (0.90)	22.0 (0.69)	--
1–2 SD (s) (*n* = 1614)	9.9 (0.47)	7.5 (0.56)	8.7 (0.44)	--	18.8 (1.14)	11.9 (0.75)	15.3 (0.62)	--	13.5 (0.75)	9.3 (0.38)	11.4 (0.42)	--
3–4 SDs (*n* = 535)	1.9 (0.18)	1.2 (0.13)	1.6 (0.10)	--	8.3 (0.53)	4.2 (0.58)	6.2 (0.37)	--	4.5 (0.46)	2.4 (0.32)	3.5 (0.33)	--
5–6 SDs (*n* = 205)	0.9 (0.16)	0.3 (0.08)	0.6 (0.08)	--	3.3 (0.33)	1.3 (0.21)	2.3 (0.19)	--	1.8 (0.24)	0.7 (0.11)	1.3 (0.14)	--
>6 SDs (*n* = 269)	1.3 (0.33)	0.7 (0.18)	1.0 (0.19)	--	4.6 (0.53)	1.5 (0.28)	3.0 (0.32)	--	2.6 (0.34)	1.0 (0.17)	1.8 (0.21)	--

^a^ Based on the weighted data. ^b^ Indicates the total number of drinking students of that category. ^c^ Rao–Scott Chi-square tests were performed to examine the differences of the whole group. ^d^ One SD contains 10 grams of pure alcohol.

**Table 4:** Prevalence, adjusted odds ratios, and 95% confidence interval of lifetime drinking, drinking in the past 30 days and drinking in the past year by socio-demographic characteristics.

Characteristics	Lifetime Drinking			Past 30 Days Drinking				Past-Year Drinking				
	*n*	%SE^a^	aOR (95% CI)^b^	*p*	*n*	%SE^a^	aOR (95% CI)^b^	*p*	*n*	%SE^a^	aOR (95% CI) ^b^	*p*
**Gender**												
Male	3497	56.0 (1.84)	1 (Reference)	--	1655	23.7 (1.29)	1 (Reference)	--	2713	43.2 (1.82)	1 (Reference)	--
Female	3358	48.9 (1.73)	0.72 (0.65, 0.78)	<0.001	1262	16.7 (0.83)	0.62 (0.54, 0.71)	<0.001	2249	33.6 (1.47)	0.64 (0.56, 0.72)	<0.001
**Grade**												
Y7	1014	39.5 (1.80)	1 (Reference)	--	389	14.0 (0.88)	1 (Reference)	--	657	25.6 (1.42)	1 (Reference)	--
Y8	1585	47.3 (2.28)	1.30 (1.06, 1.61)	0.014	641	17.6 (1.12)	1.28 (1.06, 1.53)	0.009	1113	34.1 (2.05)	1.44 (1.18, 1.75)	<0.001
Y10^r^	905	55.4 (1.70)	2.05 (1.68, 2.49)	<0.001	359	21.5 (2.03)	1.71 (1.31, 2.22)	<0.001	694	43.5 (1.74)	2.45 (2.04, 2.95)	<0.001
Y11^r^	1016	59.0 (2.36)	2.30 (1.93, 2.74)	<0.001	346	18.3 (0.97)	1.40 (1.15, 1.70)	<0.001	770	44.4 (1.73)	2.49 (2.12, 2.92)	<0.001
Y10^v^	1093	72.9 (2.78)	4.63 (3.36, 6.39)	<0.001	543	33.7 (2.68)	3.47 (2.63, 4.58)	<0.001	795	56.7 (3.52)	4.21 (3.05, 5.80)	<0.001
Y11^v^	1242	77.5 (1.43)	5.48 (4.37, 6.88)	<0.001	639	36.7 (1.69)	3.83 (3.16, 4.71)	<0.001	933	61.7 (2.33)	4.84 (3.79, 6.17)	<0.001
**Region**												
Beijing	1850	47.1 (2.68)	1 (Reference)	--	905	20.5 (1.41)	1 (Reference)	--	1176	29.7 (2.63)	1 (Reference)	--
Shanghai	1622	50.1 (2.26)	1.12 (0.89, 1.42)	0.338	674	18.8 (1.29)	0.86 (0.71, 1.03)	0.103	1271	38.3 (1.98)	1.49 (1.14, 1.93)	0.003
Guangzhou	3383	58.8 (2.60)	1.70 (1.40, 2.08)	<0.001	1338	21.6 (1.24)	1.08 (0.89,1.31)	0.455	2515	44.2 (2.41)	1.98 (1.61, 2.44)	<0.001
**Living with**												
Parents	5383	51.6 (1.76)	1 (Reference)	--	2265	19.5 (0.94)	1 (Reference)	--	3903	37.7 (1.55)	1 (Reference)	--
Mother	534	50.0 (2.09)	0.94 (0.79, 1.12)	0.464	212	18.3 (1.29)	0.93 (0.76, 1.13)	0.437	369	34.8 (2.39)	0.90 (0.73, 1.10)	0.286
Father	240	62.8 (2.59)	1.59 (1.30, 1.95)	<0.001	124	28.4 (1.98)	1.51 (1.21, 1.89)	<0.001	168	45.7 (2.71)	1.40 (1.16, 1.69)	<0.001
Others	698	59.3 (1.85)	1.15 (1.02, 1.31)	0.025	316	25.5 (1.53)	1.19 (1.01, 1.41)	0.037	522	46.8 (2.00)	1.26 (1.08, 1.47)	0.003
**SES^c^**												
Low	1340	48.0 (1.87)	1 (Reference)	--	509	17.1 (1.15)	1 (Reference)	--	917	33.1 (1.65)	1 (Reference)	--
Middle	3556	51.1 (2.19)	1.18 (1.02, 1.37)	0.026	1495	19.0 (1.06)	1.22 (1.04, 1.41)	0.012	2557	37.0 (1.94)	1.26 (1.07,1.49)	0.006
High	1959	58.2 (1.73)	1.74 (1.51, 2.01)	<0.001	913	24.7 (1.23)	1.82 (1.53, 2.15)	<0.001	1488	45.0 (1.77)	1.98 (1.65,2.36)	<0.001

^a^ Percentage by column was based on the weighted data, excluding FAS missing data. ^b^ Odds ratio was adjusted for the other variables in the model. ^c^ SES was based on the FAS score. ^r^ Indicates the regular senior high school. ^v^ Indicates the vocational senior high school.

**Table 5:** Prevalence, adjusted odds ratios, and 95% confidence interval of binge drinking and drinking until intoxication based on socio-demographic characteristics.

Characteristics	Binge Drinking				Drinking until Intoxication			
	*n*	%SE^a^	aOR (95% CI)^b^	*p*	*n*	%SE^a^	aOR (95% CI)^b^	*p*
**Gender**								
Male	510	6.6 (0.62)	1 (Reference)	--	1277	17.8 (1.17)	1 (Reference)	--
Female	225	2.9 (0.37)	0.39 (0.30, 0.50)	<0.001	979	12.2 (1.07)	0.61 (0.53, 0.69)	<0.001
**Grade**								
Y7	75	2.6 (0.43)	1 (Reference)	--	213	8.0 (0.42)	1 (Reference)	--
Y8	114	3.5 (0.34)	1.31 (0.91, 1.89)	0.147	413	12.2 (1.00)	1.56 (1.30, 1.87)	<0.001
Y10^r^	75	4.0 (0.73)	1.54 (0.91, 2.63)	0.110	265	14.4 (0.90)	1.96 (1.62, 2.36)	<0.001
Y11^r^	73	3.7 (0.47)	1.47 (0.97, 2.22)	0.070	280	13.0 (1.14)	1.74 (1.38, 2.20)	<0.001
Y10^v^	188	10.9 (1.56)	5.19 (3.30, 8.15)	<0.001	493	32.2 (3.21)	5.85 (4.34, 7.88)	<0.001
Y11^v^	210	12.1 (1.62)	6.01 (3.78, 9.55)	<0.001	592	34.9 (2.65)	6.54 (4.94, 8.65)	<0.001
**Region**								
Beijing	283	5.7 (0.66)	1 (Reference)	--	710	15.2 (1.21)	1 (Reference)	--
Shanghai	155	4.0 (0.63)	0.62 (0.47, 0.81)	<0.001	491	14.3 (1.36)	0.84 (0.66, 1.06)	0.132
Guangzhou	297	4.8 (0.57)	0.83 (0.59, 1.17)	0.288	1055	15.7 (1.44)	1.03 (0.82, 1.28)	0.816
**Living with**								
Parents	518	4.2 (0.40)	1 (Reference)	--	1690	14.0 (1.01)	1 (Reference)	--
Mother	61	4.6 (0.85)	1.07 (0.73, 1.57)	0.737	186	14.6 (1.05)	1.03 (0.88, 1.21)	0.701
Father	41	8.4 (1.05)	1.77 (1.20, 2.62)	0.004	105	25.0 (2.27)	1.88 (1.42, 2.49)	<0.001
Others	115	9.2 (1.17)	1.80 (1.38, 2.36)	<0.001	275	21.8 (2.02)	1.38 (1.21, 1.69)	0.002
**SES^c^**								
Low	121	4.1 (0.62)	1 (Reference)	--	461	14.5 (1.40)	1 (Reference)	--
Middle	336	3.8 (0.51)	1.03 (0.81, 1.31)	0.801	1137	14.1 (1.18)	1.09 (0.90, 1.32)	0.392
High	278	7.0 (0.54)	2.14 (1.58, 2.88)	<0.001	658	17.1 (1.04)	1.49 (1.26, 1.76)	<0.001

^a^ percentage by column based on the weighted data, excluding FAS missing data. ^b^ Odds ratio was adjusted for the other variables in the model. ^c^ SES was based on the FAS score. ^r^ Indicates the regular senior high school. ^v^ Indicates the vocational senior high school.

## 4. Discussion

By examining representative samples of students attending junior and senior high schools in three metropolises in China, this study provides the most current estimates of the prevalence of alcohol consumption and the characteristics of drinkers among Chinese youth. In addition, to our knowledge, this study is the first that incorporated standard drink size using a visual aid into the calculation of alcohol consumption which not only improves the estimation of quantity, but also helps with recall [27,28,29].

We found that the prevalence of drinking in senior high school students was higher than that in junior high school students; specifically, the drinking rate of vocational senior high school students was highest. In addition, a clear male and female difference regarding alcohol use were observed, except in the vocational senior high school students.

There has been no uniform definition of “drinker” used in alcohol research conducted among adolescents in China [30]; therefore, all results from previous surveys cannot be compared with each other. For example, one definition used previously defined “drinker” as an individual who drank at least one time, regardless of the quantity of alcohol consumed [31,32,33]. In another previous study, “drinker” was defined as an individual who drank a certain quantity of wine, for example, at least one glass or a small amount excluding a few sips [34,35]. Nevertheless, compared with studies using these former definitions, our findings regarding the prevalence of current drinking, which was 16.0% for all students (18.2% for boys and 13.9% for girls) in junior high school (usually in the age range of 13 to 15 years) was higher than rates reported in the GSHS fact sheet (13.0% for all students; 17.7% for boys; 8.6% for girls). Similarly, prevalence of lifetime and current drinking are higher in the present study than those were previously observed in a previous report in 2002 [32]. Notably, 21.6% of current junior high school students in Guangzhou consumed alcohol in the present study, higher than 15.7% that was reported in 2009 [36]. These figures might be interpreted as an escalation in drinking among youth in metropolises.

We also find higher rates in the present study compared with similar research in some other Asian countries, where the definition of “drinker” consisted of drinking any amount of alcoholic beverage. For example, in a 2008 Japanese study, 60.9% of 11th grade students consumed alcohol [37], which was lower than the 64.7% of our senior high school students who consumed alcohol. However, in a study of Korean students grades 6 through 12, 61.9% of males and 61.1% of females reported lifetime drinking, which was comparable to our results in China [38].

In contrast, our prevalence estimates were generally lower than those reported in western countries. For example, 70.8% of 9th to 12th grade adolescents reported any lifetime drinking in the U.S. (Centers for Disease Control and Prevention, 2011); 59.0% of 12 to 17 year olds in Mexico reported any lifetime drinking [39]; 25.9% of Asian/Pacific Islander adolescents reported drinking in the last 30 days [40]; and 82.6% of Swiss high school students 13 to 16 years of age reported drinking in the past year [41].

Although the prevalence of alcohol use among Chinese students was lower than certain western countries, an increasing trend has been observed in the past decades, as could be explained by a number of interconnected reasons. From the aspect of Chinese tradition, high school students are still considered as ‘‘children’’ by their parents and therefore they usually live with their family and obey parental orders with less rebellion at this age, yet in Western societies, this is less commonly observed. However, probably as a result of globalization, the Chinese youth are more likely to be exposed and influenced by Western culture during the past decades, which may change the Chinese tradition to some extent. The reasons for alcohol consumption in younger adolescents maybe due to the following reason: in some major Chinese festivals or parties, alcohol use is allowed and sometimes encouraged by parents, especially the grandfathers because drinking alcohol can contribute to the happiness. However, only a small amount or sips of alcoholic beverage may be allowed for most of the children.

The prevalence of lifetime alcohol use in this sample is associated with various factors, such as being male, having a higher SES, and living with fathers only. Past year drinking, current drinking, and drinking until intoxication, were associated with similar variables. The high-grade level students reported a higher frequency and quantity of consumption. There were no grade differences in the prevalence of binge drinking in the junior high school and regular senior high school students. There are numbers of interconnected reasons for this. Alcohol is considered an important tool of social communication in Chinese culture, especially for male drinkers. To some extent, this concept may also affect the social activities among young people. Some vocational senior high school individuals may start their professional career, having no limitation in alcohol consumption. Hence, there is a higher prevalence of binge drinking and drinking until intoxication among this group. Meanwhile, the prevalence showed the regional discrepancies. The following reasons may explain this. As coastal metropolis, Shanghai and Guangzhou have more trade opportunities and culture communications than Beijing, and the students' minds in these areas are more active, more like some stimulus.

Adolescent parental divorce/separation and parental history of alcohol abuse were significantly related to offspring drinking behavior [42,43,44]. In particular, males were more susceptible to binge drinking or drinking until intoxication [45,46]. Thus, adolescents who lived with their fathers may have received more encouragement to drink. In addition, adolescents with higher SES may have more pocket money to purchase drinks in pubs and parties. This is consistent with findings among British adolescents [47].

There are certain limitations in our study. First, as in most observational studies, all data were obtained through self-reports, and inaccurate recall or under-reporting may affect our results. Second, students were selected from the classes with mixed academic performance, and dropouts who were in a similar age range were excluded. If students who drop out from school are at high risk for drinking, we may have underestimated the prevalence for the whole population. Next, the study was carried out in three metropolises in China; therefore, the results cannot represent the entire country's actual adolescent drinking prevalence and patterns.

## 5. Conclusions

The information obtained from the current study among young students should draw attention to the need for school administrators as well as educational and public health agencies to collaborate in attempts to reduce alcohol availability, and to reduce drinking among young people.
